# NMR and circular dichroism data for domain 2 of the HCV NS5A protein phosphorylated by the Casein Kinase II

**DOI:** 10.1016/j.dib.2018.01.038

**Published:** 2018-01-31

**Authors:** Luiza M. Bessa, Robert Schneider, Xavier Hanoulle

**Affiliations:** University of Lille, CNRS, UMR 8576, UGSF, Unité de Glycobiologie Structurale et Fonctionnelle, F-59000 Lille, France

**Keywords:** HCV NS5A, NMR, Phosphorylation, IDP

## Abstract

The Hepatitis C Virus (HCV)^1^ nonstructural 5A protein (NS5A) is a phosphoprotein (Evans et al., 2004; Ross-Thriepland and Harris, 2014) [1], [2] composed of an N-terminal well-structured domain and two C-terminal intrinsically disordered domains (Moradpour et al., 2007; Bartenschlager et al., 2013; Badillo et al., 2017) [3], [4], [5]. So far, no precise molecular function has been identified for this viral protein (Ross-Thriepland and Harris, 2015) [6] which is required for viral replication (Tellinghuisen et al., 2008) [7]. In this article, we present datasets of NMR and circular dichroism analyses of the domain 2 of the HCV NS5A protein (NS5A-D2) phosphorylated *in vitro* by the Casein Kinase II (CKII) (Dal Pero et al., 2007; Clemens et al., 2015; Masak et al., 2014; Kim et al., 2014) [8], [9], [10], [11]. We describe the *in vitro* phosphorylation of the serine 288 (pS288) of NS5A-D2 by CKII and report the circular dichroism spectrum of the phosphorylated domain (NS5-D2_CKII). This data article also contains the ^1^H, ^15^N and ^13^C NMR chemical shift assignments (HN, N, Cα, Cβ and C’) for the phosphorylated NS5A-D2 domain, and an assigned ^1^H,^15^N-HSQC spectrum is shown. The NMR data have been acquired on an 800 MHz spectrometer. These NMR data have been used to calculate both the ^1^H,^15^N combined chemical shift perturbations (CSP) induced by the phosphorylation of pS288 and the secondary structural propensity (SSP) scores that describe the structural tendencies in this intrinsically disordered domain. The circular dichroism spectrum and the SSP scores of NS5A-D2_CKII have been compared with those of unphosphorylated NS5A-D2 [12,13].

**Specifications Table** *[please fill in right-hand column of the table below]*

**Table:** 

Subject area	*Biochemistry, Virology*
More specific subject area	*NMR structural analysis*
Type of data	*Figures, Table*
How data was acquired	*The NMR data were acquired on a Bruker AvanceIII 800 MHz spectrometer equipped with a 5 mm TXI (*^*1*^*H/*^*13*^*C/*^*15*^*N/*^*2*^*H) probe. The CD data were acquired with a Jobin Yvon-SPEX-Horiba model CD6 spectropolarimeter.*
Data format	*Analyzed*
Experimental factors	*The NS5A-D2 samples were prepared as described by Hanoulle et al.**[12]**and have been in vitro phosphorylated by CKII.*
Experimental features	*Solution state NMR experiments (2D and 3D) were acquired at 298 K on a 250 µM*^*15*^*N,*^*13*^*C-labeled-NS5A-D2_CKII sample in 20 mM sodium phosphate buffer (pH 6.4), 30 mM NaCl, 1 mM THP, 0.02% NaN*_*3*_*and 5% D*_*2*_*O. Proton chemical shifts were referenced using the methyl signal of sodium 3-trimethylsilyl-[2,2,3,3-d4]propionate at 0* *ppm.*
	*CD data were collected at 293 K on an unlabeled NS5A-D2_CKII sample (6.4 µM) in 10 mM sodium phosphate buffer (pH 6.4), 10 mM NaCl.*
Data source location	*Villeneuve d’Ascq, France, GPS: 50° 36’ 16.156” N; 3° 8’ 45.315” E*
Data accessibility	*Data are available with this article and NMR data are accessible in the Biological Magnetic Resonance data Bank (BMRB) under accession number*27270. *BMRB ID:*27270*.*http://www.bmrb.wisc.edu/data_library/summary/?bmrbId=27270

**Value of the data**

• The data showing that NS5A-D2 is phosphorylated by CKII on S288 may be useful to others researchers who study phosphorylation of the HCV NS5A protein.
• The ^1^H, ^15^N and ^13^C NMR chemical shift assignments of NS5A-D2 phosphorylated by CKII could be valuable for spectroscopists working on other phosphorylated NS5A-D2 samples and to establish comparisons.
• The CD and the ^13^Cα and ^13^Cβ chemical shifts data may be used to extract structural information, which could then be used in structure-function studies.

## 1 Data

**Fig. 1:**
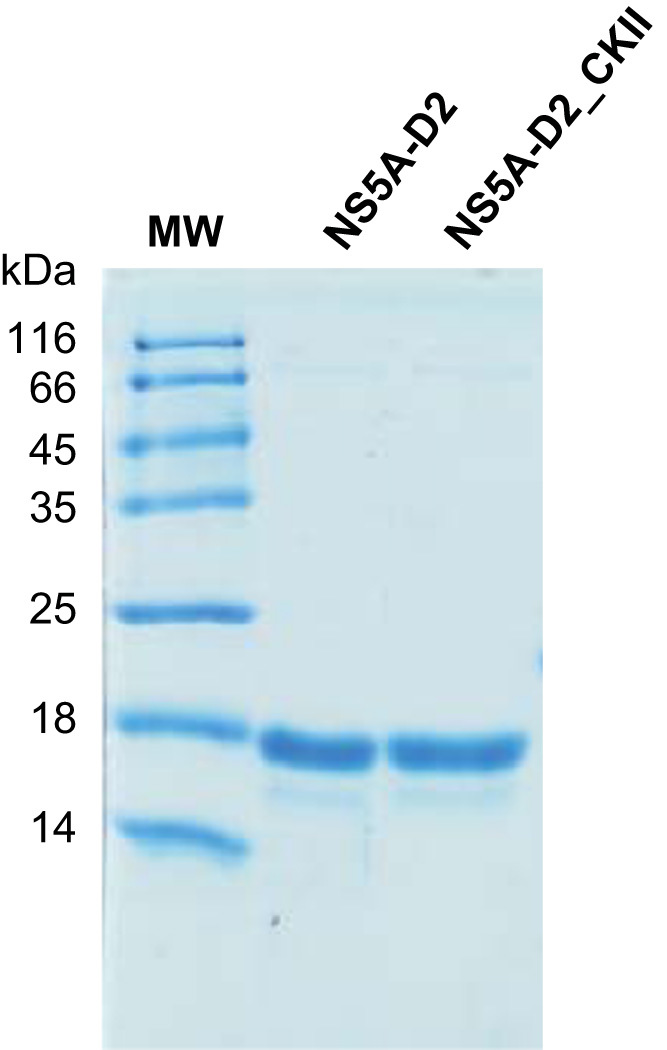
Phosphorylation of NS5A-D2 by CKII. Domain 2 of the HCV NS5A protein (NS5A-D2), expressed in *E. coli* and purified, was analyzed by 15% SDS–PAGE and stained with Coomassie blue before and after *in vitro* phosphorylation by CKII.

**Fig. 2:**
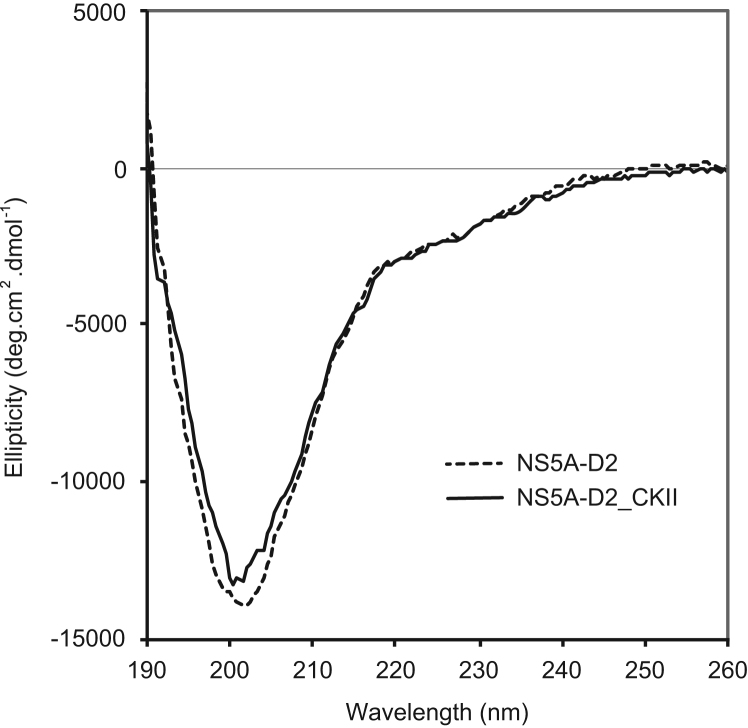
Far-UV circular dichroism analysis of NS5A-D2_CKII and comparison with NS5A-D2. The CD spectra of NS5A-D2_CKII (solid line) and NS5A-D2 (dashed line) were recorded in 10 mM sodium phosphate buffer (pH 6.4), 10 mM NaCl.

**Fig. 3:**
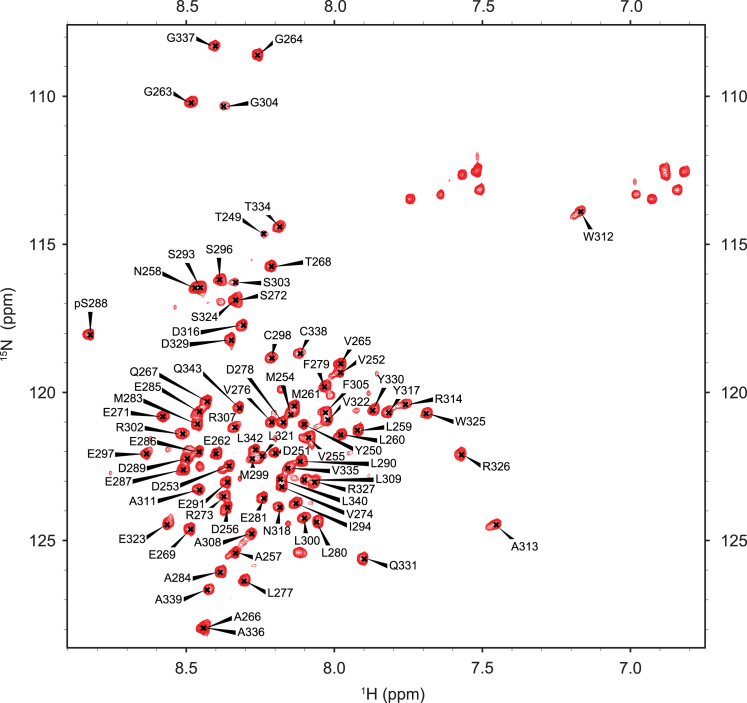
^1^H,^15^N-HSQC NMR spectrum of NS5A-D2_CKII. The spectrum has been acquired using an 800 MHz spectrometer on a 250 µM sample. Amide proton assignments listed in Table 1 are shown on the spectrum.

**Fig. 4:**
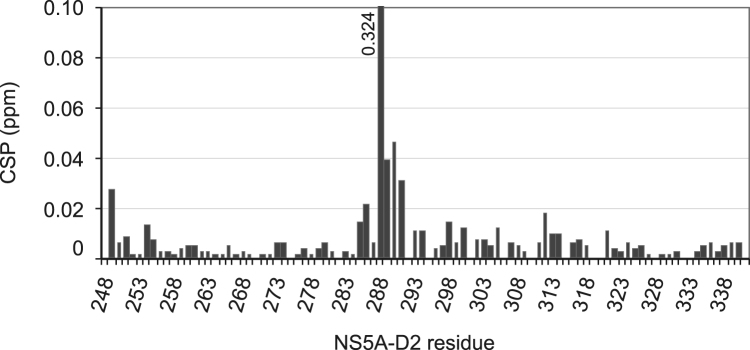
^1^H,^15^N combined chemical shift perturbations induced in NS5A-D2 by phosphorylation of S288. The CSP value of 0.324 ppm for S288 is higher than the upper limit of the graph and has thus been labeled.

**Fig. 5:**
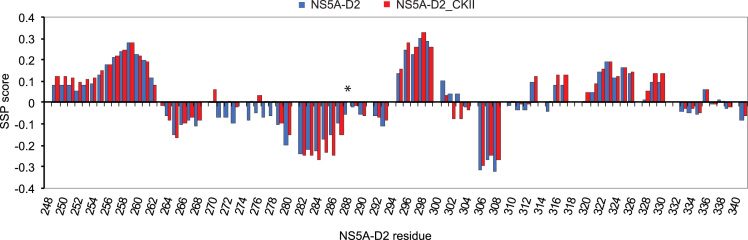
Secondary structure propensity analysis of ^13^Cα and ^13^Cβ NMR chemical shifts of NS5A-D2_CKII. Values close to 0 correspond to fully disordered residues, whereas positive and negative scores represent helical propensities and extended regions, respectively [18]. The SSP scores of NS5A-D2_CKII (in red) are compared with those of unphosphorylated NS5A-D2 (in blue). The position of the pS288 residue in NS5A-D2_CKII is highlighted by an asterisk.

We expressed the intrinsically disordered domain 2 (residues 248-341) [3], [4], [5] of the HCV NS5A protein [1], [2], which is required for viral replication [6], [7], in *Escherichia coli* and purified it as described in Hanoulle et al. [12]. Subsequently, the NS5A-D2 was *in vitro* phosphorylated by CKII (Fig. 1) [8], [9], [10], [11]. The resulting NS5A-D2_CKII sample was then analyzed by circular dichroism spectroscopy (Fig. 2) and by NMR spectroscopy. Table 1 lists the ^1^H, ^15^N and ^13^C chemical shift assignments of NS5A-D2_CKII. The annotated ^1^H-^15^N HSQC spectrum of NS5A-D2_CKII is displayed in Fig. 3. Chemical shift perturbations induced by the phosphorylation on S288 are illustrated in Fig. 4, and finally, the secondary structure propensity (SSP) scores are displayed in Fig. 5.

**Table 1:** HCV NS5A-D2_CKII NMR chemical shift assignments (BMRB ID: 27270).

**Residue**	**AA**	**HN**	**N**	**Cα**	**Cβ**	**C'**
248	N			53.23	39.06	175.07
249	T	8.24	114.66	62.23	69.62	174.13
250	Y	8.10	121.09	57.73	38.65	175.39
251	D	8.20	122.05	54.58	41.25	176.27
252	V	7.98	119.33	62.67	32.74	175.89
253	D	8.36	122.49	54.82	41.05	176.27
254	M	8.15	120.75	55.51	32.73	176.20
255	V	8.09	121.52	62.87	32.63	175.94
256	D	8.36	123.88	54.63	41.11	176.49
257	A	8.33	125.42	53.56	18.96	178.26
258	N	8.47	116.47	53.94	38.49	175.73
259	L	7.92	121.29	55.89	42.11	177.60
260	L	7.98	121.43	55.16	41.95	177.43
261	M	8.14	120.46	55.56	32.77	176.45
262	E	8.40	122.08	56.99	30.16	177.10
263	G	8.48	110.22	45.38	.	174.76
264	G	8.26	108.61	44.98	.	173.97
265	V	7.98	119.04	62.09	32.91	175.92
266	A	8.44	127.97	52.34	19.12	177.60
267	Q	8.43	120.31	55.84	29.51	176.07
268	T	8.21	115.75	61.83	70.00	174.36
269	E	8.49	124.62	54.59	29.57	174.68
270	P			63.46	32.00	177.08
271	E	8.58	120.82	56.94	30.08	176.66
272	S	8.33	116.88	58.41	63.76	174.40
273	R	8.37	123.52	55.99	30.81	176.02
274	V	8.18	123.19	60.15	32.45	174.47
275	P			63.29	32.08	176.54
276	V	8.21	121.01	62.54	32.39	176.13
277	L	8.31	126.37	54.89	42.46	176.70
278	D	8.17	121.02	54.34	41.19	175.57
279	F	8.03	119.82	57.64	39.46	175.08
280	L	8.06	124.38	54.71	42.65	176.49
281	E	8.24	123.58	54.33	29.64	174.38
282	P			63.13	32.03	176.90
283	M	8.46	121.08	55.25	33.20	175.92
284	A	8.39	126.08	52.35	19.40	177.55
285	E	8.46	120.66	56.40	30.40	176.38
286	E	8.46	122.01	56.24	30.68	176.31
287	E	8.51	122.62	56.86	30.70	176.62
288	pS	8.82	118.06	57.80	66.18	173.90
289	D	8.50	122.24	54.53	41.00	175.89
290	L	8.12	122.33	55.14	42.66	177.32
291	E	8.36	123.04	54.42	29.58	174.41
292	P			63.03	32.09	176.96
293	S	8.45	116.46	58.48	63.99	174.25
294	I	8.13	123.75	59.13	38.77	174.60
295	P			63.58	32.09	177.22
296	S	8.39	116.20	59.06	63.78	175.24
297	E	8.63	122.08	57.29	29.77	176.59
298	C	8.21	118.84	58.96	27.88	174.48
299	M	8.28	122.25	55.37	32.58	175.80
300	L	8.10	124.25	53.21	41.75	175.04
301	P			63.22	32.00	177.08
302	R	8.51	121.40	56.27	30.66	176.59
303	S	8.33	116.29	58.79	63.92	174.88
304	G	8.37	110.34	45.00	.	173.39
305	F	8.03	120.68	55.87	39.08	173.39
306	P			63.37	31.94	176.64
307	R	8.34	121.18	56.15	30.92	175.87
308	A	8.28	124.78	52.02	19.27	177.14
309	L	8.10	122.96	52.71	42.09	175.09
310	P			62.77	31.70	177.16
311	A	8.46	123.30	54.08	18.69	178.06
312	W	7.17	113.89	56.32	28.58	175.88
313	A	7.45	124.47	51.92	19.30	176.73
314	R	7.76	120.41	54.29	30.42	174.85
315	P			63.97	31.81	176.47
316	D	8.31	117.73	53.80	40.39	175.61
317	Y	7.82	120.69	58.10	38.97	174.82
318	N	8.18	123.88	50.72	39.41	171.55
319	P					
320	P			62.99	31.86	176.78
321	L	8.24	122.16	55.25	42.09	177.39
322	V	8.02	120.92	62.39	32.76	176.07
323	E	8.57	124.47	56.70	29.71	176.99
324	S	8.33	116.89	59.43	63.60	174.42
325	W	7.69	120.72	57.14	28.87	175.90
326	R	7.57	122.11	55.90	30.75	175.49
327	R	8.07	123.03	54.23	30.21	174.83
328	P			63.83	31.85	176.56
329	D	8.35	118.23	53.98	40.55	175.75
330	Y	7.87	120.61	58.40	38.97	174.91
331	Q	7.90	125.62	52.43	29.66	172.37
332	P					
333	P			63.16	31.99	176.99
334	T	8.19	114.41	61.81	69.95	174.60
335	V	8.16	122.56	62.16	32.89	175.78
336	A	8.44	127.95	52.74	19.11	178.22
337	G	8.40	108.31	45.29	.	174.08
338	C	8.12	118.69	58.32	28.26	174.05
339	A	8.43	126.68	52.29	19.21	177.26
340	L	8.18	122.94	53.20	41.83	175.29
341	P			63.11	31.82	176.81

## 2 Experimental design, materials and methods

### 2.1 *in vitro* phosphorylation

Unlabeled and doubly ^15^N,^13^C- labeled NS5A-D2 samples (from JFH1 HCV isolate, genotype 2a) were recombinantly produced in *E. coli* and then purified as described by Hanoulle et al. [12] and Dujardin et al. [14].

In a 1 ml assay, a ^15^N,^13^C-NS5A-D2 sample (140 µM) was mixed with CKII (1000 units, purchased from New England Biolabs) and 1.4 mM ATP in a buffer containing 50 mM Tris-Cl pH (7.5), 10 mM MgCl_2_, 0.1 mM EDTA, 2 mM DTT, 0.01% Brij 35. The mixture was incubated at 30 °C for 6 h. Then the buffer was exchanged using a NAP-10 column (GE Healthcare) equilibrated in 20 mM sodium phosphate buffer (pH 6.4), 30 mM NaCl, 1 mM THP, 0.02% NaN_3_ and 5% D_2_O. The phosphorylated NS5A-D2_CKII sample was concentrated up to 250 µM with a Vivaspin2 concentrator (cutoff 5 kDa). After filtration at 0.2 µm, NS5A-D2_CKII aliquots were stored at −80 °C until analyzed. Both the original NS5A-D2 and the final NS5A-D2_CKII protein samples were analyzed by SDS-PAGE on a 15% gel (Fig. 1).

### 2.2 Circular dichroism analysis

NS5A-D2 (7.3 µM) and NS5A-D2_CKII (6.4 µM) samples were analyzed at 293 K in a 1 mm-path length quartz cell using a Jobin Yvon-SPEX-Horiba model CD6 spectropolarimeter. Spectra were acquired with a step of 0.5 nm from 190 to 260 nm and an integration time of 2 s. Blank runs (with buffer only) were made before each measurement and were subtracted from the sample runs to obtain the final spectra (Fig. 2). UV intensities were expressed as the specific ellipticity per residue (per decimole of amino acid residue). Protein concentrations were determined by UV absorbance at 280 nm (molar extinction coefficient of 15595 M^−1^ cm^−1^). The CD spectrum of NS5A-D2_CKII is characteristic of mainly disordered protein with a large negative peak around 200 nm. Compared to the CD spectrum of NS5A-D2 (unphosphorylated), the only significant difference is that the negative contribution around 200 nm is slightly less pronounced.

### 2.3 NMR analyses

NMR data were acquired on a Bruker AvanceIII 800 MHz spectrometer equipped with a 5 mm TXI room-temperature probe using a Shigemi tube (sample volume 350 µL) at 298 K. Proton chemical shifts were referenced using the methyl signal of sodium 3-trimethylsilyl-[2,2,3,3-d_4_]propionate at 0 ppm. Data were acquired with Topspin2.1 software (Bruker).

All NMR datasets were collected on a single 250 µM doubly ^15^N,^13^C-labeled NS5A-D2_CKII sample in 20 mM sodium phosphate buffer (pH 6.4), 30 mM NaCl, 1 mM THP, 0.02% NaN_3_ and 5% D_2_O.

The NMR spectra collected are:

– 2D ^1^H,^15^N-HSQC spectrum, 4 scans;
– 3D ^1^H,^15^N,^13^C HNCACB, 8 scans;
– 3D ^1^H,^15^N,^13^C HNCOCACB, 8 scans;
– 3D ^1^H,^15^N,^13^C HNCO, 4 scans;
– 3D ^1^H,^15^N,^13^C HNCACO, 8 scans.

#### 2.3.1 Assignments

NMR spectra were processed with the Topspin3.2 software package (Bruker). ^1^H, ^15^N and ^13^C chemical shift assignments of NS5A-D2_CKII were performed with the product plane method developed in-house [15], using the complete NMR dataset mentioned above. The NMR data, analyzed with the software NMRFAM-SPARKY [16], showed that the residue serine 288 (S288) in NS5A-D2 is phosphorylated by CKII *in vitro* (Table 1 and Fig. 3). Backbone assignments of NS5A-D2_CKII have been deposited in the Biological Magnetic Resonance Data Bank (BMRB ID: 27270).

#### 2.3.2 Chemical shifts perturbation analysis

The chemical shifts of NS5A-D2_CKII reported in this article (BMRB ID: 27270) were compared with those of NS5A-D2 (Biological Magnetic Resonance Data Bank entry 16165) [12]. The ^1^H,^15^N combined chemical shift perturbations (CSP) were calculated using the following formula: *CSP* = √(((ΔδH)^2^ + (ΔδNH/10)^2^)/2) (adapted for IDP from [17]), where ΔδH and ΔδNH correspond to the chemical shift perturbations in the ^1^H and ^15^N dimension, respectively. The CSP values are illustrated in Fig. 4. The residue with the highest CSP value (0.324 ppm) corresponds to the serine 288 which is phosphorylated in the NS5A-D2_CKII sample. The other residues that exhibit CSP values larger than 0.15 ppm are mainly located around the phosphorylation site and correspond to the NS5A-D2 region encompassing residues 285–291, but also include the T249 and the W312 residues.

#### 2.3.3 Secondary Structure Propensity analysis

Experimental ^13^C chemical shifts of ^13^Cα and ^13^Cβ nuclei of NS5A-D2_CKII were analyzed with the Secondary Structure Propensity (SSP) software [18] to calculate a score illustrating structural propensity for each NS5A-D2_CKII residue. The plot of the SSP data along the NS5A-D2 sequence is shown in Fig. 5. SSP scores close to 0 correspond to fully disordered residues, whereas positive and negative scores represent helical propensities and extended regions, respectively. The low SSP scores in Fig. 5 show that even in the presence of the pS288, NS5A-D2 is still mainly disordered with two main regions exhibiting partial alpha-helical structuration (residues 252–261 and 295–299) and two others showing partial extended conformation (residues 279–288 and 306–308). SSP values of NS5A-D2_CKII were then compared with those obtained from the unphosphorylated NS5A-D2 sample that we have previously reported [13]. Overall, SSP data in the absence and in the presence of the phosphorylation at position 288 are very similar. Only subtle differences were detected in three NS5A-D2 regions: directly around the phosphorylation site (residues 282–287), in the region of residues 271–278, and in residues 301–304 (Fig. 5). Just prior to the pS288 site (residues 282–287) the propensity to adopt an extended conformation is slightly higher than in the absence of phosphorylation. This is probably due to charge repulsion phenomena between the negatively charged phosphate moiety of pS288 and the carboxyl groups in the side chains of E285, E286 and E287. In the region of residues 271–278, NS5A-D2_CKII seems to be more flexible when phosphorylated, as illustrated by SSP scores close to 0. In residues 301–304, the SSP scores shift from low positive to low negative values, showing a slight change in the conformation of this region upon phosphorylation.

## References

[1] Evans M.J., Rice C.M., Goff S.P. (2004). Phosphorylation of hepatitis C virus nonstructural protein 5A modulates its protein interactions and viral RNA replication. Proc. Natl. Acad. Sci. USA.

[2] Ross-Thriepland D., Harris M. (2014). Insights into the complexity and functionality of hepatitis C virus NS5A phosphorylation. J. Virol..

[3] Moradpour D., Penin F., Rice C.M. (2007). Replication of hepatitis C virus. Nat. Rev. Microbiol..

[4] Bartenschlager R., Lohmann V., Penin F. (2013). The molecular and structural basis of advanced antiviral therapy for hepatitis C virus infection. Nat. Rev. Microbiol..

[5] Badillo A., Receveur-Brechot V., Sarrazin S., Cantrelle F.-X., Delolme F., Fogeron M.-L., Molle J., Montserret R., Bockmann A., Bartenschlager R., Lohmann V., Lippens G., Ricard-Blum S., Hanoulle X., Penin F. (2017). Overall structural model of NS5A protein from Hepatitis C Virus and modulation by mutations confering resistance of virus replication to cyclosporin A. Biochemistry.

[6] Ross-Thriepland D., Harris M. (2015). Hepatitis C virus NS5A: enigmatic but still promiscuous 10 years on!. J. Gen. Virol..

[7] Tellinghuisen T.L., Foss K.L., Treadaway J.C., Rice C.M. (2008). Identification of residues required for RNA replication in domains II and III of the hepatitis C virus NS5A protein. J. Virol..

[8] Dal Pero F., Di Maira G., Marin O., Bortoletto G., Pinna L.A., Alberti A., Ruzzene M., Gerotto M. (2007). Heterogeneity of CK2 phosphorylation sites in the NS5A protein of different hepatitis C virus genotypes. J. Hepatol..

[9] Clemens K., Yeh C.-Y., Aizenman E. (2015). Critical role of casein kinase 2 in hepatitis C NS5A-mediated inhibition of Kv2.1 K(+) channel function. Neurosci. Lett..

[10] Masaki T., Matsunaga S., Takahashi H., Nakashima K., Kimura Y., Ito M., Matsuda M., Murayama A., Kato T., Hirano H., Endo Y., Lemon S.M., Wakita T., Sawasaki T., Suzuki T. (2014). Involvement of hepatitis C virus NS5A hyperphosphorylation mediated by casein kinase I-α in infectious virus production. J. Virol..

[11] Kim S., Jin B., Choi S.H., Han K.-H., Ahn S.H. (2014). Casein kinase II inhibitor enhances production of infectious genotype 1a hepatitis C virus (H77S). PLoS One.

[12] Hanoulle X., Badillo A., Wieruszeski J.M., Verdegem D., Landrieu I., Bartenschlager R., Penin F., Lippens G. (2009). Hepatitis C virus NS5A protein is a substrate for the peptidyl-prolyl cis/trans isomerase activity of cyclophilins A and B. J. Biol. Chem..

[13] Rosnoblet C., Fritzinger B., Legrand D., Launay H., Wieruszeski J.-M., Lippens G., Hanoulle X. (2012). Hepatitis C Virus NS5B and host cyclophilin A share a common binding site on NS5A. J. Biol. Chem..

[14] Dujardin M., Madan V., Montserret R., Ahuja P., Huvent I., Launay H., Leroy A., Bartenschlager R., Penin F., Lippens G., Hanoulle X. (2015). A proline-tryptophan turn in the intrinsically disordered domain 2 of NS5A protein is essential for Hepatitis C Virus RNA replication. J. Biol. Chem..

[15] Verdegem D., Dijkstra K., Hanoulle X., Lippens G. (2008). Graphical interpretation of Boolean operators for protein NMR assignments. J. Biomol. NMR.

[16] Lee W., Tonelli M., Markley J.L. (2015). NMRFAM-SPARKY: enhanced software for biomolecular NMR spectroscopy. Bioinformatics.

[17] Garrett D.S., Seok Y.-J., Peterkofsky A., Clore G.M., Gronenborn A.M. (1997). Identification by NMR of the binding surface for the histidine-containing phosphocarrier protein HPr on the N-terminal domain of enzyme I of the *Escherichia coli* phosphotransferase system. Biochemistry.

[18] Marsh J.A., Singh V.K., Jia Z., Forman-Kay J.D. (2006). Sensitivity of secondary structure propensities to sequence differences between alpha- and gamma-synuclein: implications for fibrillation. Protein Sci..

